# Cerebrospinal Fluid Amyloid‐β Biomarkers Predict Future Hemorrhage in Patients with Cerebral Amyloid Angiopathy

**DOI:** 10.1002/ana.78241

**Published:** 2026-04-27

**Authors:** Philipp Arndt, Malte Pfister, Valentina Perosa, Hendrik Mattern, Melis Tas, Lara Holzheimer, Johanna Engel, Marc Dörner, Marwa Al‐Dubai, Cornelia Garz, Eya Khadhraoui, Sebastian J. Müller, Patrick Müller, Sven G. Meuth, Katja Neumann, Stefanie Schreiber

**Affiliations:** ^1^ Department of Neurology Otto‐von‐Guericke University Magdeburg Germany; ^2^ German Center for Neurodegenerative Diseases (DZNE) within the Helmholtz Association Magdeburg Germany; ^3^ J. Philip Kistler Stroke Research Center, Massachusetts General Hospital/Harvard Medical School Boston MA USA; ^4^ Biomedical Magnetic Resonance, Faculty of Natural Sciences Otto‐von‐Guericke University Magdeburg Germany; ^5^ Center for Behavioral Brain Sciences Magdeburg Germany; ^6^ Department of Consultation‐Liaison‐Psychiatry and Psychosomatic Medicine University Hospital Zurich, University of Zurich Zurich Switzerland; ^7^ Department of Neuroradiology Otto‐von‐Guericke University Magdeburg Germany; ^8^ Department of Cardiology Otto‐von‐Guericke University Magdeburg Germany; ^9^ Department of Neurology Heinrich‐Heine‐University Düsseldorf Germany

## Abstract

**Objective:**

Accurately predicting future hemorrhagic events in patients with cerebral amyloid angiopathy (CAA) remains a major clinical challenge. It is unknown whether cerebrospinal fluid (CSF) biomarkers of amyloid‐beta (Aβ) pathology are associated with increased hemorrhage risk in this population.

**Methods:**

We analyzed consecutive patients meeting Boston criteria version 2.0 for probable CAA with CSF Aβ data obtained during diagnostic workup. The primary outcome was incident intracranial hemorrhage, including lobar intracerebral, convexity subarachnoid, and non‐traumatic subdural hemorrhage. Secondary outcomes were ischemic stroke and all‐cause mortality. Associations between low CSF Aβ biomarkers and outcomes were analyzed using Cox proportional hazards models, adjusted for disseminated cortical superficial siderosis, and prior intracerebral hemorrhage.

**Results:**

Among 109 patients (median age: 77 years, 42% female), 16 (15%) experienced incident intracranial hemorrhage and 11 (10%) incident ischemic stroke during a median follow‐up of 2.93 years (interquartile range: 1.43–5.03). In multivariate Cox regression models low CSF Aβ biomarkers were independently associated with incident intracranial hemorrhage (Aβ_40_: hazard ratio: 8.04; [95% CI: 2.43–26.59], *p* < 0.001; Aβ_42_: hazard ratio 7.10; [95% CI: 1.58–32.00], *p* = 0.011). CSF Aβ biomarkers were not associated with incident ischemic strokes and all‐cause mortality. A composite risk score integrating low CSF Aβ biomarkers and hemorrhagic imaging features identified a high‐risk subgroup with 78% hemorrhage incidence (7/9 patients) and a no‐risk group without events (0/42 patients).

**Interpretation:**

Low CSF Aβ biomarkers are independently associated with future symptomatic hemorrhage in CAA patients. A composite risk score may support individualized risk stratification and guide clinical decision‐making. ANN NEUROL 2026;100:391–399

Cerebral amyloid angiopathy (CAA) is a common small vessel disease characterized by deposition of amyloid‐beta (Aβ) in cortical and leptomeningeal vessels. Progressive vascular amyloid deposition compromises vessel integrity and promotes rupture, ultimately leading to intracranial hemorrhage (ICrH).^1^ Because of that patients with CAA are at increased risk for future symptomatic lobar intracerebral hemorrhage (ICH), convexity subarachnoid hemorrhage (SAH), and subdural hemorrhage (SDH).^2^, ^3^

Although hemorrhagic neuroimaging markers at baseline such as lobar ICH (odds ratio: 2.08) and disseminated cortical superficial siderosis (cSS) (hazard ratio [HR]: 4.28) are established risk indicators for future hemorrhage in CAA,^4^, ^5^ predicting future symptomatic hemorrhage remains a major clinical challenge in patients with CAA. Fluid biomarkers reflecting upstream pathophysiological mechanisms such as impaired Aβ clearance or even exacerbation of Aβ retention could potentially identify patients at risk before overt hemorrhagic injury occurs. Reduced levels of cerebrospinal fluid (CSF) Aβ_42_ and Aβ_40_ are indicative of Aβ accumulation in the brain and able to differentiate patients with CAA from controls. Furthermore, low Aβ_40_ differentiates patients with Alzheimer's disease (AD) from CAA patients, indicating a more specific marker for amyloid vasculopathy.^6^

Although a small retrospective study suggested that low CSF Aβ_42_ levels may be associated with the presence of ICH and cSS at presentation,^7^ it remains unknown whether CSF Aβ biomarkers can prospectively predict incident hemorrhagic events in patients with CAA. Recent evidence from a lobar ICH cohort at our center demonstrated that low CSF Aβ biomarker levels were associated with a higher risk of recurrent hemorrhage and aided to distinguish probable CAA from other underlying etiologies.^8^ Establishing a prospective link between CSF Aβ biomarkers and future symptomatic hemorrhage in diverse CAA cohorts could, therefore, provide a disease‐specific tool for individualized risk stratification and secondary prevention. To address this unmet need, we investigated whether CSF Aβ_40_ and Aβ_42_ levels were independently associated with future ICrH in a well‐characterized cohort of patients with probable CAA.

## Methods

### Study Population and Baseline Data Collection

**FIGURE 1:**
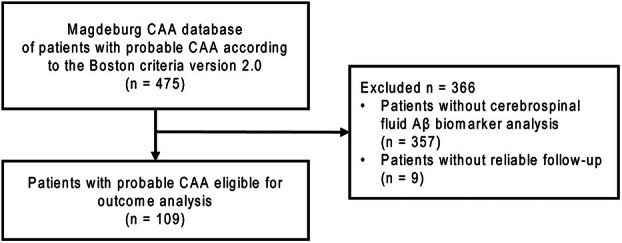
Study flowchart.

We included n = 109 consecutive patients from our prospectively curated database on CAA between April 2013 and January 2025 at the Department of Neurology, Otto‐von‐Guericke University Magdeburg. Inclusion criteria were: (1) diagnosis of probable CAA according to the Boston criteria version 2.0,^9^ (2) available CSF Aβ biomarker analysis from lumbar puncture performed as part of the diagnostic workup, and (3) available follow‐up information on cerebrovascular events, confirmed by neuroimaging, and mortality. Lumbar puncture and CSF analysis was offered to all patients with CAA as part of the diagnostic workup, based on clinical indication (cognitive decline, exclusion of differential diagnoses, or to support diagnosis). We excluded CAA patients without reliable follow‐up data (n = 9) (Fig 1). Cognitive status was determined based on clinical assessment and available neuropsychological reports.

### Outcome Analysis

The primary outcome of interest was incident ICrH, including spontaneous lobar ICH, non‐aneurysmal convexity SAH, and non‐traumatic SDH. Secondary outcomes of interest were (1) ischemic stroke, and (2) death of any cause. Follow‐up data were obtained through a systematic review of multiple overlapping sources, including our prospectively maintained CAA database, electronic medical records (discharge summaries and follow‐up outpatient reports), institutional radiologic databases, or structured telephone or in‐person interviews with patients or their relatives. Incident outcome events were defined as clinically symptomatic cerebrovascular events and required confirmation by neuroimaging.

### Magnetic Resonance Imaging Acquisition and Analysis

For analysis, clinical 3 T magnetic resonance imaging (MRI) (Skyra, n = 51) or 1.5 T MRI (Sola, n = 57; both from Siemens Heathineers, Erlangen, Germany) was used to quantify cSS, lobar microbleeds (MB), white matter hyperintensities (WMH), lacunes, and perivascular spaces (PVS) according to the Standards for Reporting Vascular Changes on Neuroimaging criteria^10^ by 1 trained investigator (M.P., more than 3 years of experience) blinded to CSF data. The following sequences were used: T2*‐weighted gradient‐recalled echo for cSS and MB, T2‐weighted fluid‐attenuated inversion recovery (FLAIR) for WMH and lacunes, T2‐weighted turbo spin echo for PVS in the basal ganglia (BG) and centrum semiovale (CSO). Presence and number of MB or lacunes were assessed according to the Microbleed Anatomic Rating Scale.^11^ WMH in deep and periventricular regions were rated according to the Fazekas scale and specific WMH patterns as recently proposed.^12^ cSS was defined as curvilinear hypointensities following the cortical surface distinct from vessels, and was assessed according to a validated scale: absent, focal (restricted to 1 sulcus or ≤3 immediately adjacent sulci), or disseminated (affecting ≥2 non‐adjacent sulci or ≥4 immediately adjacent sulci). High degree of PVS (number of PVS >20) in either BG or CSO were rated as recently proposed.^13^

### CSF Aβ Biomarkers

CSF samples were collected, centrifuged at 4°C, aliquoted, and stored at −80°C until analysis. CSF samples were processed according to standard laboratory quality‐control procedures; samples not meeting laboratory quality criteria for biomarker analysis were not included. CSF Aβ_40_, Aβ_42_, and pTau_181_ levels were measured using enzyme‐linked immunosorbent assay kits (until December 2019: Innotest, Innogenetics, Ghent, Belgium; n = 72) or automated immunoassays (from January 2020: LUMIPULSE G600 II, Fujirebio, Japan; n = 36). To account for methodological variability between assay platforms, CSF biomarker values were standardized using z‐transformation within each assay group, ensuring comparability across methods.^14^ Z‐scores were calculated using the mean and standard deviation (SD) of the respective assay‐specific CAA subgroup, such that each transformed value reflects the number of SD from the subgroup mean. The median interval between the initial MRI and lumbar puncture was 5 days (interquartile range [IQR]: 1–25 days). For patients with lobar ICH, the median interval between ICH and lumbar puncture was 8 days (IQR: 0–89).

### Protocol Approvals

The local Clinical Ethics Committee approved this retrospective study (no. 07/17, addendum 11/2021). Informed consent was obtained from all subjects contacted in this study to evaluate longitudinal progression. The study was performed in accordance with the relevant local and national guidelines and regulations.

### Statistical Analysis

Continuous variables were expressed as median (IQR) or mean (SD), as appropriate, and categorical variables as proportions. Group comparisons between patients with and without incident hemorrhage were conducted using the *t* test, Mann–Whitney *U*‐test, χ^2^ test, or Fisher's exact test as appropriate.

Receiver operating characteristic (ROC) analysis of assay‐standardized z‐scores was performed to assess the discriminative ability of CSF Aβ biomarkers for incident hemorrhage, with optimal cut‐off values determined by maximizing Youden's J statistic. Sensitivity and specificity values reflect performance at these thresholds. These ROC‐derived thresholds were used to define low CSF Aβ levels for prognostic analyses.

Survival time was calculated from the date of initial presentation to the first occurrence of an outcome event of interest or the last available follow‐up without that event. For patients experiencing multiple outcome events, time was censored at the first outcome. Patients were also censored at the time of death if no prior outcome occurred. To assess the association between CSF Aβ biomarkers and the risk of future ICrH, univariate Cox proportional hazards models were first applied using ROC‐derived cut‐offs for low CSF Aβ_40_ and Aβ_42_ levels. Corresponding event‐free survival was visualized using Kaplan–Meier curves. Multivariable Cox regression was then performed to assess the independent predictive value of each biomarker, adjusting for prior lobar ICH and disseminated cSS. Given the limited number of outcome events, multivariable Cox models were restricted to these 2 prespecified predictors to avoid model overfitting and over adjustment for closely related clinical manifestations. HR with 95% confidence intervals (CI) were reported. A prespecified sensitivity analysis was conducted in subgroups of patients with and without prior lobar ICH, adjusted for disseminated cSS. Secondary outcome analyses examined associations of CSF Aβ biomarkers with (1) ischemic stroke and (2) all‐cause mortality. All Cox regression analyses were performed to assess etiologic associations rather than to develop or validate a prognostic prediction model. A significance threshold of *p* < 0.05 was applied for all analyses. Statistical analyses were performed using IBM SPSS Statistics 28.0 and R 4.4.2, with Kaplan–Meier curves generated in GraphPad Prism 10.0.

## Results

Among 475 consecutive patients with probable CAA in our institutional database, 109 (23%) were included in the outcome analysis (Fig 1). The median age was 77 years (IQR: 72–80), 46 patients (42%) were female, and 30 (28%) initially presented with symptomatic lobar ICH. All patients fulfilled the Boston criteria version 2.0 for probable CAA and 99 patients (91%) additionally fulfilled the Boston criteria version 1.5 for probable CAA. Compared with excluded patients, the CSF cohort was enriched for patients with cognitive impairment, seizures, and hypertension, whereas lobar ICH was underrepresented (Table S1). Mini–Mental State Examination (MMSE) scores were available in n = 58 (53%) patients and were more frequently obtained in patients with clinically documented cognitive impairment (n = 40/60 [67%], median 23, IQR: 17–24) than in those without (n = 18/49 [37%], median 28, IQR: 26–29), which may result in overrepresentation of lower MMSE scores.

### CSF Biomarkers and Risk of Future ICrH

During a median follow‐up of 2.93 years (IQR: 1.43–5.03 years; 346 patient‐years), 16 (15%) patients experienced 20 ICrH (18 lobar ICH, 1 convexity SAH, 1 non‐traumatic SDH; 5.8 per 100 patient‐years), and 11 (10%) patients experienced an ischemic stroke (3.2 per 100 patient‐years). During follow‐up, 43 (39%) patients died.

Patients who developed an ICrH during follow‐up had significantly lower CSF Aβ_42_ (*p* < 0.001) and Aβ_40_ (n = 0.005) levels at baseline than those who did not. Demographics, vascular risk factors, and CSF pTau_181_ levels were similar between groups, while known predictors (eg, disseminated cSS [p = 0.003], prior lobar ICH [p = 0.005], and transient focal neurological episodes [p = 0.025]) were more prevalent among those with incident hemorrhage. Other neuroimaging markers showed no relevant differences (Table 1). ROC analysis showed that both Aβ markers discriminated moderately well between patients with and without incident hemorrhage (Aβ_42_: AUC: 0.79; 95% CI: 0.69–0.89; sensitivity 81%, specificity 65%, cut‐off −0.447; Aβ_40_: AUC: 0.70; 95% CI: 0.54–0.87; sensitivity 50%, specificity 95%, cut‐off −1.143). The corresponding ROC‐derived thresholds were subsequently used to define low CSF Aβ levels for Cox regression analyses.

**TABLE 1:** Key Characteristics of Patients with and without Hemorrhage during Follow‐Up

Characteristics	Whole CAA cohort n = 109	Hemorrhage during follow‐up n = 16	No hemorrhage during follow‐up n = 93	*p*
Age	77 (72–80)	75 (73–78)	77 (71–81)	0.235
Female	46 (42%)	8 (50%)	38 (41%)	0.494
Hypertension	90/102 (88%)	14 (88%)	76/86 (88%)	1.000
Type 2 diabetes	24/102 (24%)	3 (19%)	21/86 (24%)	0.757
Dyslipidemia	36/102 (35%)	9 (56%)	27/86 (32%)	0.056
Lobar intracerebral hemorrhage	30 (28%)	9 (56%)	21 (23%)	**0.005**
Convexity SAH	13 (12%)	3 (19%)	10 (11%)	0.402
TFNE	9 (8%)	4 (25%)	5 (5%)	**0.025**
CAA‐related inflammation	4 (4%)	0 (0%)	4 (4%)	1.000
Prior ischemic stroke	32 (31%)	4 (25%)	28 (33%)	0.770
Prior seizure	30 (28%)	4 (25%)	26 (28%)	1.000
Gait disturbances	41 (38%)	4 (25%)	37 (40%)	0.403
Cognitive impairment	60 (55%)	8 (50%)	52 (56%)	0.660
Mild cognitive impairment	24 (22%)	5 (31%)	19 (20%)	0.335
Dementia	36 (33%)	3 (19%)	33 (36%)	0.255
Mini mental state examination^a^	23 (19–26)	26 (23–26)	23 (17–26)	0.079
Focal cSS	11 (10%)	2 (13%)	9 (10%)	0.663
Disseminated cSS	15 (14%)	6 (38%)	9 (10%)	**0.003**
Number of lobar MB	5 (2–16)	8 (1–19)	5 (2–16)	0.997
Multispot WMH pattern	79 (73%)	14 (88%)	66 (72%)	0.231
WMH Fazekas scale	5 (4–6)	5 (4–6)	5 (4–6)	0.487
Severe CSO PVS	99 (91%)	13 (81%)	86 (93%)	0.163
Severe BG PVS	37 (34%)	2 (13%)	35 (38%)	0.083
Presence of lacunes	42 (39%)	5 (31%)	37 (40%)	0.517
CSF Aβ_40_ [z‐score]	0.00 (1.00)	−0.62 (1.00)	0.14 (0.97)	**0.005**
CSF Aβ_42_ [z‐score]	0.00 (1.00)	−0.71 (0.36)	0.15 (1.02)	**<0.001**
CSF pTau_181_ [z‐score]	0.00 (1.00)	0.16 (1.04)	0.00 (1.01)	0.531
Low CSF Aβ_40_	13 (12%)	8 (50%)	5 (5%)	**<0.001**
Low CSF Aβ_42_	46 (42%)	13 (81%)	33 (36%)	**<0.001**

*Note:* Significant comparisons are highlighted bold. Abbreviations: Aβ = amyloid‐beta; BG = basal ganglia; CAA = cerebral amyloid angiopathy; CSF = cerebrospinal fluid; CSO = centrum semiovale; cSS = cortical superficial siderosis; HR = hazard ratio; MB = microbleed; PVS = perivascular spaces; SAH = subarachnoid hemorrhage; TFNE = transient focal neurological episodes; WMH = white matter hyperintensities.

^a^ MMSE data were only available for n = 58 (53%) CAA patients (ie, for n = 11 [69%] with hemorrhage and for n = 47 [50%] without hemorrhage during follow‐up).

**FIGURE 2:**
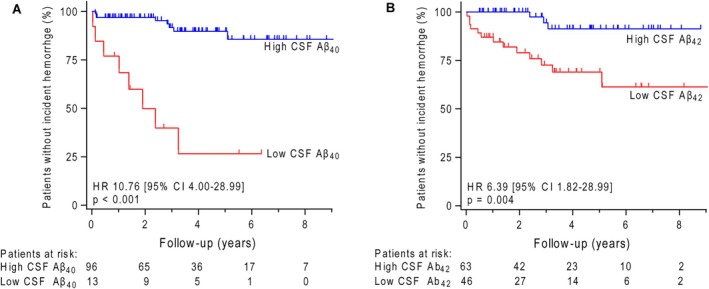
Kaplan–Meier curves of event‐free survival according to cerebrospinal fluid (CSF) amyloid‐beta (Aβ) biomarker levels. A and B show time to incident intracranial hemorrhage for CSF Aβ_40_ and Aβ_42_, respectively, including lobar intracerebral hemorrhage, non‐aneurysmal convexity subarachnoid hemorrhage, and non‐traumatic subdural hemorrhage. Groups were stratified by low versus high CSF biomarker levels using receiver operating characteristic (ROC)‐derived cut‐off values. Hazard ratios (HR), 95% confidence intervals (CI), and *p*‐values are based on univariate Cox regression models. [Color figure can be viewed at www.annalsofneurology.org]

In multivariable Cox regression adjusted for disseminated cSS and prior lobar ICH, low CSF Aβ_40_ (HR: 8.04; 95% CI: 2.43–26.59, n < 0.001) and low Aβ_42_ (HR: 7.10; 95% CI: 1.58–32.00, n = 0.011) were independently associated with incident hemorrhage. Disseminated cSS (HR: 4.81, n = 0.006) and prior lobar ICH (HR: 9.77, n < 0.001) were also independent predictors (Fig 2, Table 2).

**TABLE 2:** Cox Proportional Hazards Regression Analyses of CSF Aβ Biomarkers and Incident Intracranial Hemorrhage during Follow‐Up in Patients with CAA

	Univariate models		Multivariate model	
	HR [95% CI]	*p*	HR [95% CI]	*p*
Low CSF Aβ_40_	10.76 [4.00–28.99]	**<0.001**	8.04 [2.43–26.59]	**<0.001**
Low CSF Aβ_42_	6.39 [1.82–22.41]	**0.004**	7.10 [1.58–32.00]	**0.011**
Disseminated cSS	5.79 [2.08–16.14]	**<0.001**	4.81 [1.56–14.88]	**0.006**
Prior lobar ICH	4.62 [1.71–12.49]	**0.003**	9.77 [3.16–30.18]	**<0.001**

*Note*: Among the 109 patients included in this subgroup analysis, 16 experienced an incident hemorrhagic event during follow‐up. Univariate and multivariable models report HR with 95% CI. Significant associations are highlighted bold.

Abbreviations: Aβ = amyloid‐beta; CI = confidence interval; CSF = cerebrospinal fluid; cSS = cortical superficial siderosis; HR = hazard ratio; ICH = intracerebral hemorrhage.

In sensitivity analyses, we evaluated the predictive value of CSF biomarkers in (1) patients with and (2) without lobar ICH (Tables 3 and 4). In n = 30 patients with prior lobar ICH, univariate Cox regression revealed that both low CSF Aβ biomarkers were associated with incident hemorrhages (9 patients with incident event; CSF Aβ_42_: HR: 5.88; 95% CI: 1.44–24.06, *p* = 0.014; CSF Aβ_40_: HR: 20.05; 95% CI: 3.63–111.73, *p* < 0.001). Disseminated cSS was not a significant predictor in this subgroup (*p* = 0.857) and was subsequently not entered into the multivariate model. In the multivariate model, including both CSF Aβ biomarkers, only CSF Aβ_40_ remained significant (HR: 10.44; 95% CI: 1.45–75.35, *p* = 0.020). In n = 79 patients without prior lobar ICH, univariate Cox regression revealed that low CSF Aβ_40_ remained associated with future hemorrhage (7 patients with incident event; HR: 12.95; 95% CI: 2.87–58.43, *p* < 0.001). Effect size and significance were unchanged after adjustment for disseminated cSS (HR: 12.83; 95% CI: 2.08–79.22, *p* = 0.006). Estimates for CSF Aβ_42_ from univariate models were imprecise, with wide CIs, reflecting the small number of hemorrhagic events in this subgroup. The variable was subsequently not included into the multivariate model.

**TABLE 3:** Cox Proportional Hazards Regression Analyses of CSF Aβ Biomarkers and Incident Intracranial Hemorrhage during Follow‐Up in Patients with CAA and Prior Lobar Intracerebral Hemorrhage

	Univariate models		Multivariate model	
	HR [95% CI]	*p*	HR [95% CI]	*p*
Low CSF Aβ_40_	20.05 [3.63–111.73]	**<0.001**	10.44 [1.45–75.35]	**0.020**
Low CSF Aβ_42_	5.88 [1.44–24.06]	**0.014**	2.66 [0.44–16.03]	0.286
Disseminated cSS	1.16 [0.24–5.60]	0.857		

*Note*: Among the 30 patients included in this subgroup analysis, 9 experienced an incident hemorrhagic event during follow‐up. Univariate and multivariable models report HR with 95% CI. Significant associations are highlighted bold.

Abbreviations: Aβ = amyloid‐beta; CI = confidence interval; CSF = cerebrospinal fluid; cSS = cortical superficial siderosis; HR = hazard ratio; ICH = intracerebral hemorrhage.

**TABLE 4:** Cox Proportional Hazards Regression Analyses of CSF Aβ Biomarkers and Incident Intracranial Hemorrhage during Follow‐Up in Patients with CAA and without Prior Lobar Intracerebral Hemorrhage

	Univariate models		Multivariate model	
	HR [95% CI]	*p*	HR [95% CI]	*p*
Low CSF Aβ_40_	12.95 [2.87–58.43]	**<0.001**	12.83 [2.08–79.22]	**<0.001**
Low CSF Aβ_42_	81.01 [0.18–37198.6]	0.160		
Disseminated cSS	21.65 [4.73–99.15]	**<0.001**	23.10 [3.67–145.21]	**<0.001**

*Note*: Among the 79 patients included in this subgroup analysis, 7 experienced an incident hemorrhagic event during follow‐up. Univariate and multivariable models report HR with 95% CI. Significant associations are highlighted bold.

Abbreviations: Aβ = amyloid‐beta; CI = confidence interval; CSF = cerebrospinal fluid; cSS = cortical superficial siderosis; HR = hazard ratio.

As secondary outcomes, we assessed incident ischemic strokes and mortality. In univariate cox regression models, both CSF Aβ biomarkers were not associated with incident ischemic stroke (*p* > 0.20) and mortality (*p* > 0.30) (Table S2).

### Composite Risk Score

**FIGURE 3:**
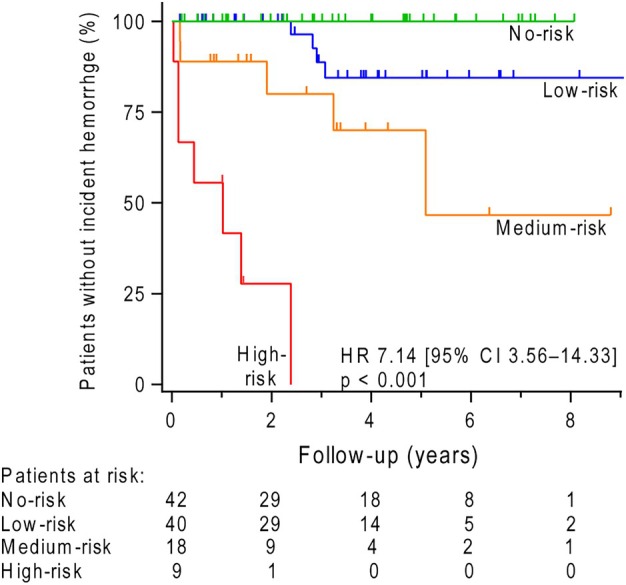
Kaplan–Meier curves of event‐free survival according to the composite risk score. The panel shows time to incident intracranial hemorrhage for the composite risk score summing the 4 independent predictors: low cerebrospinal fluid (CSF) Aβ_40_, low CSF Aβ_42_, disseminated cortical superficial siderosis (cSS), and prior lobar intracerebral hemorrhage. Patients were classified as no (0 factors), low (1 factor), medium (2 factors), or high risk (3–4 factors). Hazard ratios (HR), 95% confidence intervals (CI), and *p*‐values are based on univariate Cox regression models. [Color figure can be viewed at www.annalsofneurology.org]

To explore combined risk patterns, we developed an exploratory composite hemorrhage risk score by summing the 4 independent predictors: low CSF Aβ_40_, low CSF Aβ_42_, disseminated cSS, and prior lobar ICH (1 point for the presence of each factor). Patients were classified as no (0 factors), low (1 factor), medium (2 factors), or high risk (3–4 factors). The composite risk score was strongly associated with incident hemorrhage in univariable Cox regression (HR: 7.14; 95% CI: 3.56–14.33, *p* < 0.001), indicating a stepwise increase in hemorrhagic risk, with events in 0 of 42 (0%) no‐risk, 4 of 40 low‐risk (10%), 5 of 18 (28%) medium‐risk, and 7 of 9 (78%) high‐risk patients (Fig 3). All variable combinations and corresponding outcomes are listed in Table S3. High‐risk patients displayed imaging features of hemorrhagic activity and reduced CSF Aβ levels, reflecting manifest bleeding tendency and advanced Aβ vasculopathy. The composite score showed high apparent discrimination for incident hemorrhage (AUC: 0.88; 95% CI: 0.80–0.96), exceeding that of the combination of disseminated cSS and lobar ICH alone (AUC: 0.77; 95% CI: 0.64–0.89). As the score was derived and evaluated in the same dataset without internal validation, these discrimination estimates should be considered descriptive.

## Discussion

Our study demonstrates that reduced CSF Aβ_40_ and Aβ_42_ concentrations are independently associated with future symptomatic ICrH in patients with probable CAA, even when accounting for established predictors. These biomarkers may reflect vascular Aβ pathology and offer prognostic value beyond conventional imaging. Notably, low CSF Aβ_40_ also remained informative among patients with and without prior ICH. Based on these findings, we developed a composite risk score integrating biofluid and imaging markers, which demonstrated high discriminatory accuracy and identified a subgroup in which no hemorrhagic events occurred during follow‐up.

The assessment of hemorrhage risk is critical for the prognosis of patients, as well as for physicians who balance the risk and benefit of anticoagulation, antiplatelet therapy, and other interventions for secondary prevention.^15^ Well‐established risk factors such as prior lobar hemorrhage and disseminated cSS reflect accumulated vascular damage, but may appear late in the disease course.^1^, ^4^, ^5^, ^16^ Their absence does, however, not reliably indicate low risk, especially in early‐stage CAA, memory clinic populations, and the emerging anti‐amyloid therapies.^17^, ^18^ In contrast, CSF Aβ biomarkers may capture upstream pathophysiological processes earlier.

CSF Aβ biomarkers are well established in AD and reflect the burden of parenchymal Aβ plaques, which also indicate a predisposition to concurrent CAA. Consequently, they have been associated with the diagnosis of CAA in comparison to controls or individuals with mixed location hemorrhages and provide an alternative to identify CAA when MRI is unavailable or to strengthen the suspected diagnosis.^6^, ^19^, ^20^ Their mechanistic role in CAA and particularly hemorrhagic risk warrants, however, further exploration. For example, it is not known whether CSF Aβ biomarkers inform beyond diagnostic categorization about vascular Aβ burden. Our findings support the hypothesis that reduced CSF Aβ levels reflect aggravation of impaired amyloid clearance, which is mechanistically linked to vessel wall fragility and rupture as suggested from accumulating evidence.^1^ This aligns with observations in Dutch‐type hereditary CAA, where symptomatic individuals exhibited 30% lower CSF Aβ_40_ and 21% lower Aβ_42_ levels than presymptomatic carriers, further supporting a threshold effect in CSF biomarkers preceding overt hemorrhage.^16^ Our data support the hypotheses that very low CSF Aβ_40_ levels, observed in only a small subset of patients, provide a high rule‐in specificity in predicting incident hemorrhage. CSF Aβ_40_ is suggested as a more CAA‐specific biomarker because of its preferential deposition in vessel walls, but is also influenced by overall Aβ production. Therefore, in AD diagnostics, the Aβ_42/40_ ratio is routinely used to correct for such interindividual variability and to enhance specificity for parenchymal Aβ pathology.^21^

Importantly, unlike AD diagnostics, standardized clinical cut‐offs for CSF Aβ_40_ are currently not established. In our study, “low” Aβ_40_ and Aβ_42_ were defined empirically using outcome‐based ROC analyses on assay‐standardized z‐scores (Youden's J) and, therefore, should be interpreted as cohort‐specific risk stratification thresholds rather than universally applicable laboratory cut‐offs. The threshold for low Aβ_42_ (−0.45 SD) identified a larger proportion of patients and showed higher sensitivity for future hemorrhage, whereas the low Aβ_40_ threshold (−1.14 SD) was present in a small minority and provided high specificity, thereby identifying a particularly high‐risk subgroup. Clinically, these biomarkers are best interpreted as complementary to established MRI markers. CSF Aβ_42_ might indicate advanced amyloid pathology, and very low Aβ_40_ may serve as a “red flag” in selected patients. However, it should be acknowledged that thresholds require site‐specific calibration and external validation before routine implementation. Our findings are supported and contextualized by 2 recent studies exploring CSF biomarkers in CAA. One demonstrated that CAA patients initially presenting with symptomatic ICH exhibit significantly lower CSF Aβ_40_ levels compared to those without hemorrhagic presentation, whereas CSF Aβ_42_ and tau biomarkers remained unchanged.^22^ Our study extends these observations by demonstrating that very low CSF Aβ_40_ not only characterizes hemorrhagic CAA phenotypes at presentation, but also prospectively predicts incident symptomatic hemorrhage. A second study reported an absence of incident hemorrhagic events during follow‐up in n = 16 CAA patients without CSF Aβ pathology (ie, not meeting established CSF Aβ_42_ threshold for AD diagnostics).^23^ This is in line with the high sensitivity of CSF Aβ_42_ in our cohort and the reported no‐risk group if established imaging predictors are additionally recognized. Furthermore, they showed that patients with isolated Aβ pathology experienced higher prospective hemorrhagic risk than patients with concomitant tau pathology.^23^ This contrasts with our data, where CSF pTau_181_ levels had no prognostic value.

The combined use of CSF Aβ biomarkers with established hemorrhagic risk factors from neuroimaging enhances risk stratification and is urgently needed in CAA. The ability to identify patients with no or low hemorrhage risk could enable more personalized management, including tailored discussions on antithrombotic therapy for prevalent comorbidities. Conversely, identifying patients at very high risk could justify intensified surveillance or inclusion in preventive trials. However, given the limited number of outcome events, our multivariable analyses should be interpreted as exploratory and require external validation and assay harmonization before routine clinical implementation.

Notably, decreased CSF Aβ levels represent one of the earliest detectable markers of CAA, preceding radiological signs of hemorrhage.^1^, ^16^ In our study, low CSF Aβ_40_ together with disseminated cSS emerged as a significant predictor of future hemorrhage even in patients without a prior history of ICH. However, given the low event rate in this subgroup, future prospective studies are needed to validate these findings and determine whether reductions in CSF Aβ levels mark a true tipping point toward first symptomatic ICrH in early‐stage CAA. In summary, these findings strengthen the growing evidence that integrating Aβ fluid biomarkers into a comprehensive diagnostic framework enhances precision in cerebrovascular disease and refines individual risk assessment for optimal clinical care.

## Limitations and Future Directions

Several limitations of this study warrant consideration. The retrospective design, small number of incident events, and reliance on patients who underwent lumbar puncture introduce selection bias, limiting generalizability. Lumbar puncture is contraindicated in patients with high intracranial pressure because of hemorrhage, which may restrict its applicability to some patients with suspected CAA. Additionally, recent ICrH may limit the reliability of CSF Aβ measurements because of potential blood admixture. Specifically, patients with cognitive impairment or seizures were overrepresented in this cohort, whereas those presenting with lobar ICH were underrepresented, reflecting both clinical indications and contraindications for lumbar puncture. However, the lobar ICH incidence in our cohort (5.2 per 100 patient‐years) and the associated effect size of disseminated cSS (adjusted HR: 4.81) were comparable to those reported in large CAA cohorts, supporting external plausibility while acknowledging differences in cohort composition.^4^ Given the limited number of events relative to the number of predictors, Cox regression analyses may have led to unstable point estimates and wide CIs, limiting the reliability and interpretability of these exploratory findings. Although models were adjusted for established imaging markers, additional clinical factors such as blood pressure control and antithrombotic medication could not be included in multivariable analyses because of the limited number of outcome events and the risk of model overfitting, but should be recognized in larger studies. Furthermore, death was treated as a censoring event in cause‐specific Cox models and formal competing risk analyses were not performed. To address these limitations, future studies should externally validate our findings in larger, multicenter prospective cohorts, assess the impact of competing mortality using dedicated competing risk approaches, and explore alternative, less invasive biomarkers, such as Aβ levels from blood‐based assays. The rapid evolution of highly accurate diagnostic blood‐based biomarkers in AD, particularly phosphorylated tau at position 217, offers a promising model for extending molecular diagnostics to CAA.^24^, ^25^

## Author Contributions

P.A. and S.S. contributed to the conception and design of the study; P.A., C.G., and M.P. contributed to the acquisition and analysis of data; all authors contributed to drafting the text or preparing the figures.

## Potential Conflicts of Interest

Nothing to report.

## Supporting information


**Table S1.** Comparison of baseline characteristics between included CAA patients who underwent CSF analysis and those who did not and were subsequently excluded.
**Table S2.** Cox proportional hazards regression analyses of CSF Aβ biomarkers and (i) incident ischemic stroke, and (ii) death during follow‐up in patients with CAA.
**Table S3.** Combination patterns of risk variables and corresponding hemorrhage rates. Distribution of all observed combinations of 4 independent predictor variables (low CSF Aβ40, low CSF Aβ42, disseminated cSS, and prior lobar ICH), the resulting composite risk score (0–4), assigned risk group, and corresponding number of patients and hemorrhagic events during follow‐up.

## Data Availability

The data that support the findings of this study are available from the corresponding author on reasonable request.
