# Supplementary material for: Cerebrospinal Fluid Amyloid‐β Biomarkers Predict Future Hemorrhage in Patients with Cerebral Amyloid Angiopathy
Source: Ann Neurol. 2026 Apr 27;100(2):391–9. doi: 10.1002/ana.78241 (PMC13387976; doi:10.1002/ana.78241)
Supplement: Supplementary file 1 — Table S1. Comparison of baseline characteristics between included CAA patients who underwent CSF analysis and those who did not and were subsequently excluded. Table S2. Cox proportional hazards regression analyses of CSF Aβ biomarkers and (i) incident ischemic stroke, and (ii) death during follow‐up in patients with CAA. Table S3. Combination patterns of risk variables and corresponding hemorrhage rates. Distribution of all observed combinations of 4 independent predictor variables (low CSF Aβ40, low CSF Aβ42, disseminated cSS, and prior lobar ICH), the resulting composite risk score (0–4), assigned risk group, and corresponding number of patients and hemorrhagic events during follow‐up. [file ANA-100-391-s001.docx]

**Supplementary material**

**Supplementary Table 1. Comparison of baseline characteristics between included CAA patients who underwent CSF analysis and those who did not and were subsequently excluded.**

| **Characteristics** | **Excluded CAA patients n = 366** | **Included CAA patients n = 109** | **p-value** |
| --- | --- | --- | --- |
| Age | 76 (70-81) | 77 (72-80) | 0.320 |
| Female | 165 (45%) | 46 (42%) | **0.673** |
| Hypertension | 259 (71%) | 90/102 (88%) | **< 0.001** |
| Type 2 diabetes | 84 (23%) | 24/102 (24%) | 0.870 |
| Dyslipidemia | 123 (34%) | 36/102 (35%) | 0.840 |
| Lobar intracerebral hemorrhage | 170 (46%) | 30 (28%) | **0.001** |
| Prior ischemic stroke | 99 (27%) | 32 (31%) | 0.725 |
| Prior seizure | 58 (16%) | 30 (28%) | **0.009** |
| Gait disturbances | 72/183 (39%) | 41 (38%) | 0.866 |
| Cognitive impairment | 55 (15%) | 60 (55%) | **< 0.001** |

**Supplementary Table 2. Cox proportional hazards regression analyses of CSF Aβ biomarkers and (i) incident ischemic stroke, and (ii) death during follow-up in patients with CAA.**

Among the 109 patients included in this subgroup analysis, 11 experienced an incident ischemic stroke and 43 died during follow-up. Univariate models report hazard ratios (HR) with 95% confidence intervals (CI).

|  | **Univariate models** | | | |
| --- | --- | --- | --- | --- |
|  | **Ischemic stroke** | | **Death** | |
|  | *HR [95% CI]* | *p-value* | *HR [95% CI]* | *p-value* |
| Low CSF Aβ_40_ | 2.35 [0.49-11.20] | 0.284 | 1.56 [0.65-3.71] | 0.318 |
| Low CSF Aβ_42_ | 0.84 [0.25-2.89] | 0.786 | 0.86 [0.46-1.60] | 0.634 |

Abbreviations: Aβ, amyloid-beta; CI, confidence interval; CSF, cerebrospinal fluid; HR, hazard ratio.

**Supplementary Table 3. Combination patterns of risk variables and corresponding hemorrhage rates.** Distribution of all observed combinations of four independent predictor variables (low CSF Aβ40, low CSF Aβ42, disseminated cSS, and prior lobar ICH), the resulting composite risk score (0–4), assigned risk group, and corresponding number of patients and hemorrhagic events during follow-up.

| **Low  CSF Aß40** | **Low  CSF Aß42** | **Disse-minated  cSS** | **Prior lobar ICH** | **Score  (0-4)** | **Risk Group** | **Patients (n)** | **Hemorrhages (n)** |
| --- | --- | --- | --- | --- | --- | --- | --- |
| 1 | 1 | 1 | 1 | 4 | High | 1 | 1 |
| 0 | 1 | 1 | 1 | 3 | High | 2 | 1 |
| 1 | 1 | 0 | 1 | 3 | High | 3 | 3 |
| 1 | 1 | 1 | 0 | 3 | High | 3 | 2 |
| 0 | 1 | 1 | 0 | 2 | Medium | 4 | 2 |
| 0 | 0 | 1 | 1 | 2 | Medium | 4 | 0 |
| 0 | 1 | 0 | 1 | 2 | Medium | 5 | 1 |
| 1 | 1 | 0 | 0 | 2 | Medium | 5 | 2 |
| 1 | 0 | 0 | 0 | 1 | Low | 1 | 0 |
| 0 | 0 | 1 | 0 | 1 | Low | 1 | 0 |
| 0 | 0 | 0 | 1 | 1 | Low | 15 | 3 |
| 0 | 1 | 0 | 0 | 1 | Low | 23 | 1 |
| 0 | 0 | 0 | 0 | 0 | No | 42 | 0 |
